# Rapid Purification and Characterization of Mutant Origin Recognition Complexes in *Saccharomyces cerevisiae*

**DOI:** 10.3389/fmicb.2016.00521

**Published:** 2016-04-18

**Authors:** Hironori Kawakami, Eiji Ohashi, Toshiki Tsurimoto, Tsutomu Katayama

**Affiliations:** ^1^Department of Molecular Biology, Graduate School of Pharmaceutical Sciences, Kyushu UniversityFukuoka, Japan; ^2^Department of Biology, Faculty of Science, Kyushu UniversityFukuoka, Japan

**Keywords:** DNA replication, ORC, protein purification, recombinant protein, *S. cerevisiae*

## Abstract

Purification of the origin recognition complex (ORC) from wild-type budding yeast cells more than two decades ago opened up doors to analyze the initiation of eukaryotic chromosomal DNA replication biochemically. Although revised methods to purify ORC from overproducing cells were reported later, purification of mutant proteins using these systems still depends on time-consuming processes including genetic manipulation to construct and amplify mutant baculoviruses or yeast strains as well as several canonical protein fractionations. Here, we present a streamlined method to construct mutant overproducers, followed by purification of mutant ORCs. Use of mammalian cells co-transfected with conveniently mutagenized plasmids bearing a His tag excludes many of the construction and fractionation steps. Transfection is highly efficient. All the six subunits of ORC are overexpressed at a considerable level and isolated as a functional heterohexameric complex. Furthermore, use of mammalian cells prevents contamination of wild-type ORC from yeast cells. The method is applicable to wild-type and at least three mutant ORCs, and the resultant purified complexes show expected biochemical activities. The rapid acquisition of mutant ORCs using this system will boost systematic biochemical dissection of ORC and can be even applied to the purification of protein complexes other than ORC.

## Introduction

Purification of mutant proteins from overproducing cells constituted a milestone in biochemistry to analyze proteins of interest. However, construction of mutant overproducers and purification of the mutant proteins from these overproducers under native conditions depend on time-consuming processes, especially when the activities of high-order protein complexes are to be examined *in vitro*.

**Figure 1:**
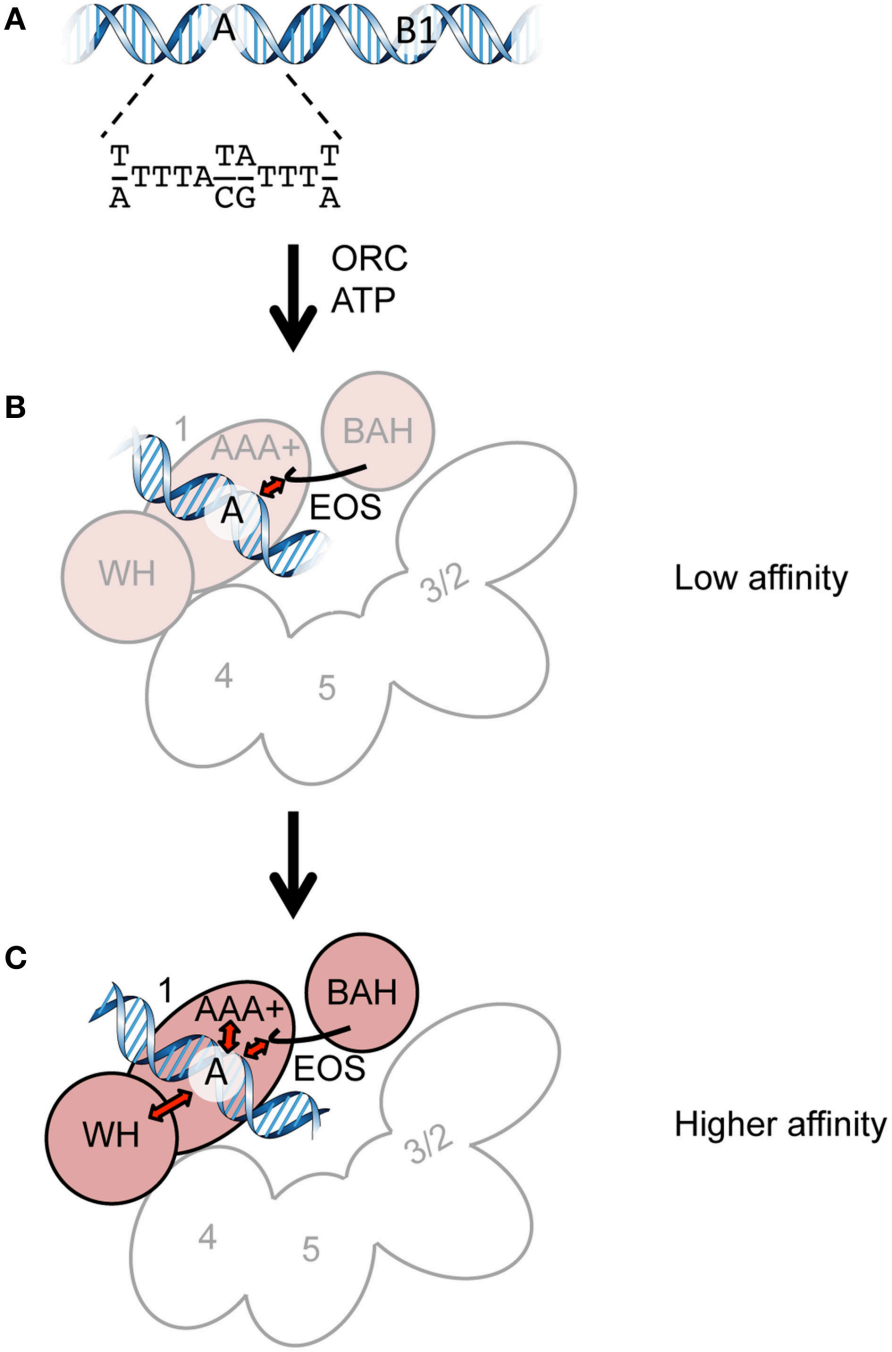
**Cartoons summarizing ORC-ARS binding. (A)** The ORC-binding regions of ARS. The A and B1 elements and the ARS consensus sequence are indicated. **(B)** Direct recognition of the A element by EOS with a low affinity. Orc1 and Orc2/3/4/5 are shown in pink and white, respectively. Orc6 and the WH domains of Orc2/3/4/5 are omitted for clarity. **(C)** Recognition of the A element via EOS and other domains in a mutually supportive manner with a higher affinity. The initial interaction of EOS with the A element leads to additional interactions with other domains (possibly AAA+ and WH domains), resulting in high-affinity binding. The ORC subunits are shown as in panel **(B)**.

The origin recognition complex (ORC), consisting of Orc1/2/3/4/5/6, is one such protein complex (Duncker et al., 2009; Kawakami and Katayama, 2010; Li and Stillman, 2012). ORC binds to eukaryotic chromosomal replication origins in an ATP-dependent manner to recruit Cdc6, Cdt1, and the MCM2-7 helicase core onto double-stranded DNA (Boos et al., 2012; Bell and Kaguni, 2013; Yardimci and Walter, 2014; Tognetti et al., 2015). In the budding yeast *Saccharomyces cerevisiae*, replication origins are called autonomously replicating sequences (ARSs). ARSs bear two major functional elements, namely, the essential A element containing the ARS consensus sequence and the stimulatory B elements (Figure 1). The A and B1 elements are essential for ORC binding. All ORC subunits except for Orc6 are highly conserved among eukaryotes and belong to the AAA+ (ATPases associated with a variety of cellular activities) superfamily, although only Orc1 and Orc5 bind to ATP. ORC ATPase activity is repressed by ARS DNA *in vitro* and thought to ensure timely recruitment of the MCM2-7 helicase. Orc1/2/3/4/5 also bear one or two winged-helix DNA-binding motifs at the C-terminus. Orc1 bears an extension at the N-terminus called the BAH (bromo-adjacent homology) domain that binds to transcription-related proteins. The linker region between BAH and AAA+ bears a highly conserved, basic residue-rich motif called the eukaryotic origin sensor (EOS) that solely and directly scans the essential element in ARS with a low affinity to achieve high-affinity binding of the ORC hexamer to ARS (Figure 1; Kawakami et al., 2015). Elimination of one subunit (except for Orc6) from the ORC hexamer abolishes high-affinity binding of ORC to ARS (Lee and Bell, 1997), suggesting that purification of the entire ORC complex rather than individual subunits is important to analyze the biological functions of ORC.

Purification of ORC was first achieved from wild-type yeast cells (Bell and Stillman, 1992) and later revised using overproducing cells. One approach is to use insect cells co-transfected with three types of baculoviruses carrying two of the six ORC subunits (Bell et al., 1995; Fujita et al., 1998; Sun et al., 2012, 2013; Samel et al., 2014). Another approach is to construct a yeast strain with inducible promoters (Remus et al., 2009; Hizume et al., 2013). Although both approaches can be used to analyze wild-type ORC, site-directed mutagenesis of ORC is time-consuming with the aforementioned systems because the former requires construction and amplification of baculoviruses and the latter requires yeast genetics for integration of the overproducing cassettes into the yeast genome. Both approaches require several classical fractionation processes, which are rate-limiting, to purify several mutant ORCs. In the latter system, endogenous wild-type ORC might contaminate the mutant ORC fractions, which would falsely indicate that the mutants have hypomorphic phenotypes. Recently, an improved method was developed to overcome part of these problems in purification of ORC containing a site-specifically mutated Orc4 subunit (Frigola et al., 2013; Coster et al., 2014). In this method, CBP (calmodulin-binding peptide)-tagged Orc1, mutant Orc4, and intact Orc2/3/5/6 were co-expressed in a yeast strain in which endogenous wild-type Orc4 is Flag-tagged. Affinity chromatography via the CBP-tagged Orc1 co-purifies both wild-type and mutant Orc4; the contaminated wild type is then excluded by immunodepletion during further purification including HPLC-based fractionation steps. Although this approach may be applicable to purification of ORC hexamers containing any of the other mutant subunits, extra tagging(s) to endogenous *ORC* loci for immunodepletion, as well as the above-mentioned iterated gene integrations and still laborious purification steps, should be required to obtain each of mutant ORCs desired. Indeed, mutant ORC hexamers purified to date are limited to those with point mutations in the representative AAA+ domains (Klemm et al., 1997; Klemm and Bell, 2001; Bowers et al., 2004; Speck et al., 2005; Speck and Stillman, 2007; Coster et al., 2014) and deletion of the Orc1 BAH (Frigola et al., 2013; Hizume et al., 2013).

The novel method described herein uses co-transfection of mammalian cells with conveniently mutagenized plasmids bearing a His tag (shorter than a CBP tag), which excludes many of the construction and fractionation steps. HPLC-based procedure in this method is kept minimum. Furthermore, use of mammalian cells prevents contamination of wild-type ORC from yeast cells, which eliminates immunodepletion step(s) of wild type mentioned above. Purified wild-type and mutant ORCs using this system showed expected biochemical activities; therefore, the rapid acquisition of mutant ORCs using this highly versatile system will boost systematic biochemical dissection of ORC and can be even applied to purify protein complexes other than ORC.

## Materials and methods

### Buffers

Buffer H′ contained 50 mM Hepes-KOH (pH 7.6), 0.02% NP-40, 10% [v/v] glycerol, 1 mM benzamidine, 2.5 μg/ml pepstatin A, 0.1 mg/ml bacitracin, and 0.5 mM PMSF. Lysis buffer was the same as buffer H′ except that 0.3% NP-40, 400 mM KCl, 5 mM β-mercaptoethanol, and 17.5 mM imidazole were included. Wash buffer was the same as lysis buffer except that 0.02% NP-40 was added. Elution buffer was the same as wash buffer except that 500 mM imidazole was added. H/0.2 and H/0.4 were the same as buffer H′ except that 200 and 400 mM KCl were added, respectively, as well as 1 mM each of EDTA, EGTA, and DTT. Buffer K contained 45 mM Hepes-KOH [pH 7.6], 4.5 mM magnesium acetate, 140 mM KCl, and 9% [v/v] glycerol.

### Plasmids

The mammalian overexpression vector version (ver.) 3–5 (Uno et al., 2012) was a gift from Dr. Hisao Masai. pHK106 (*ORC1*), pHK107 (*ORC2*), pHK108 (*ORC3*), pHK109 (*ORC4*), pHK110 (*ORC5*), and pHK111 (*ORC6*) were constructed by sequence- and ligation-independent cloning (SLIC; Li and Elledge, 2007) so that PCR-amplified *ORC1*/*2*/*3*/*4*/*5*/*6* fragments could be inserted between the Kozak sequence and the HpaI site of ver. 3–5 and that the His and HA tags of ver. 3–5 were eliminated. pHK118 was constructed by QuikChange site-directed mutagenesis (Agilent) using pHK106 so that a hexahistidine tag could be appended to the C-terminus of Orc1 with a linker (WNLYFQS; identical to a TEV recognition sequence). pHK122 was constructed by Gibson assembly (Gibson et al., 2009) using pHK118 and pACYCDuet-1 (Novagen) so that the p15A*ori*-*cat* cassette could replace the ColE1*ori*-*bla* cassette of pHK118. pHK123 (*orc1 K362A-His*) and pHK124 (*orc1 R367A-His*) were constructed by QuikChange mutagenesis using pHK118. pKSEO212 is a plasmid for expression of mAG-6His-tagged hRad9. The *hRad9* cDNA fragment was inserted into BamHI-XbaI sites of CSII-EF-MCS-mAG-6His-Claspin-3Flag plasmid (Uno and Masai, 2011), replacing the Claspin-3Flag with hRad9. The cloned genes and flanking regions of the abovementioned plasmids were verified by sequencing.

### Overexpression of ORC subunits

Overexpression in 293T cells was performed by transfection using the PEI method as described previously (Uno et al., 2012) except that 0.26 μg of each plasmid per 10 cm plate was transfected, unless specified otherwise.

### Western blotting

Monoclonal antibodies against Orc1 (SB13) and His tag were gifts from Dr. Bruce Stillman. Chemiluminescent signals were detected in an ImageQuant LAS 4010 imager (GE Healthcare).

### Quantitative biochemistry

An electrophoretic mobility shift assay using Cy5-labeled *ARS1* DNA was performed as described previously (Kawakami et al., 2015). ORC ATPase activity, which is repressible by ARS, was assayed as described previously (Klemm et al., 1997) with slight modifications. Briefly, a 76-bp segment of wild-type *ARS1* (position 818–893) or a mutant (–ACS) was amplified by PCR using primers HK301 (CTTGCCTGCAGGCCTTTTG) and HK302 (ATCTTTACATCTTGTTATTTTACAGATTTTATG). The amplified DNA was incubated with 0.4 pmol of ORC, 15 μM [α-^32^P]ATP, and 3 pmol of a 290-bp GC-rich competitor (Speck et al., 2005) for 45 min at 25°C in 10 μl of buffer K. The reaction was stopped by adding 5 μl of 2% SDS. The resultant radiolabeled ADP was quantified by thin layer chromatography, followed by phosphoimaging as described previously (Kawakami et al., 2005).

## Results and discussion

### Construction of *S. cerevisiae* ORC-overproducing plasmids for a mammalian expression system

**Figure 2:**
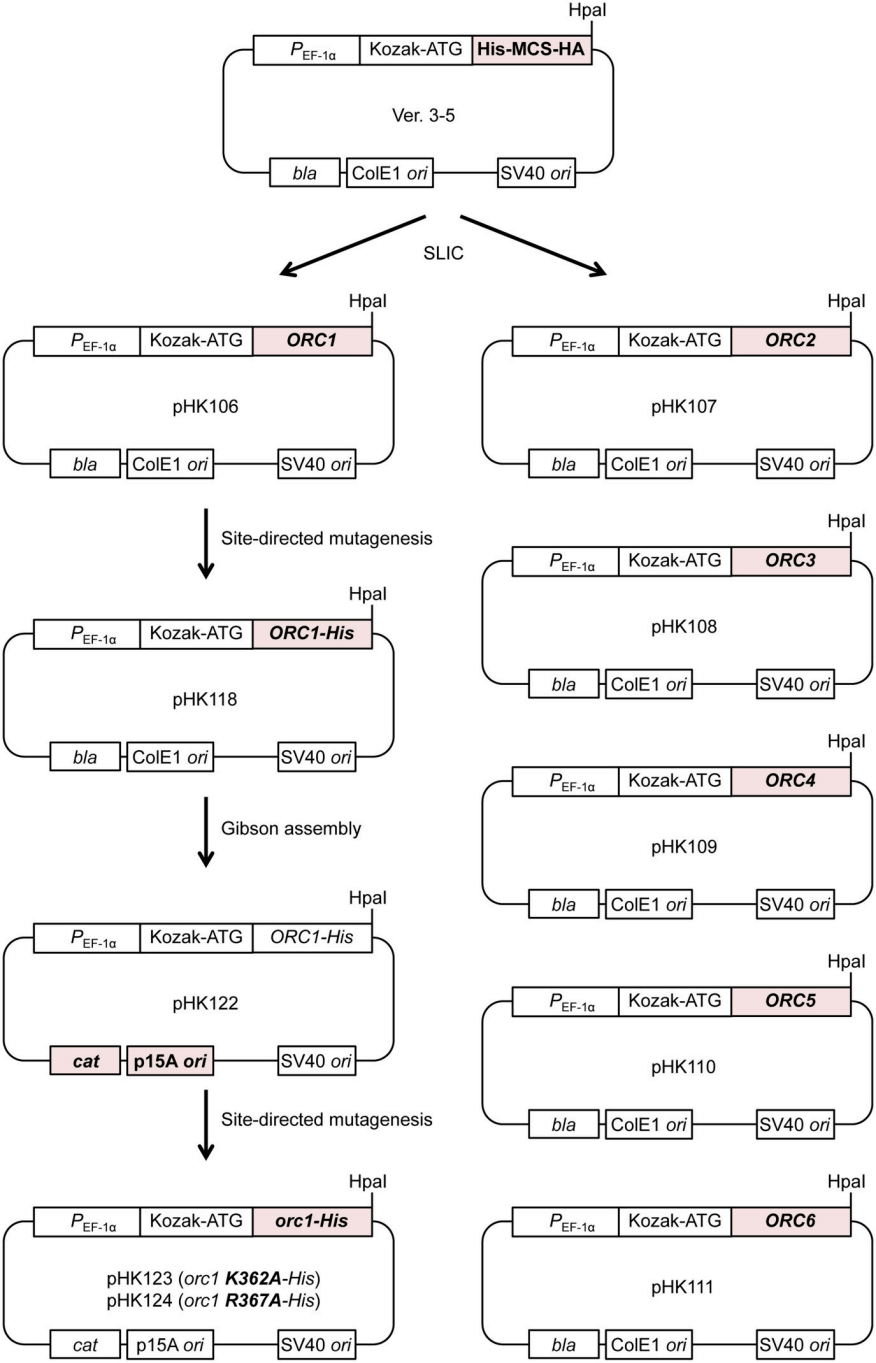
**Construction of ORC-overproducing plasmids**. All the pHK plasmids used in this study are derivatives of ver. 3–5. See text for details.

To overproduce *S. cerevisiae* ORC in mammalian cells, we modified the ver. 3–5 vector for transfection, which was originally developed to overexpress proteins that are not overexpressed well in bacterial or insect cells (Figure 2; Uno and Masai, 2011; Uno et al., 2012). Ver. 3–5 is a shuttle vector carrying *P*_*EF*−1α_, a Kozak sequence, a His tag, a multiple cloning site, and an HA tag. Ver. 3–5 also bears an SV40 origin, which could help to maintain the vector episomally in mammalian cells expressing the large T antigen, such as 293T cells. To maintain the plasmid in *Escherichia coli*, ver. 3–5 also bears *bla* and ColE1 *ori*. We first cloned one of the *ORC1/2/3/4/5/6* genes into ver. 3–5 so that the N-terminal His tag and C-terminal HA tag of the vector were eliminated, yielding pHK106 through to pHK111. By site-directed mutagenesis, a hexahistidine sequence with a linker was appended just before the stop codon of *ORC1*, yielding pHK118. This tag, consisting of 13 amino acids, is shorter than a CBP-TEV tag previously used for Orc1 tagging (Frigola et al., 2013). Addition of a short tag, such as His_12_ or His-Strep II, to the C-terminus of Orc1 does not affect Orc1 function *in vivo* (Kawakami et al., 2015). We noticed that introduction of certain *orc1* mutations into pHK118 by site-directed mutagenesis was unsuccessful. Because the same mutation could be introduced into another *ORC1* plasmid under the control of the native *ORC1* promoter using the same mutagenic primers (Kawakami et al., 2015), one plausible idea is that leaky expression of Orc1 from pHK118 may be extremely toxic in *E. coli* cells only when a certain *orc1* mutation is introduced. Because Orc1 solely binds to the ARS sequence via a domain termed EOS (Kawakami et al., 2015), similar binding to a similar sequence in the *E. coli* genome may be affected by the mutation and interfere with a certain cellular process *in vivo*. Alternatively, adverse interactions of the Orc1 AAA+ domain with other AAA+ proteins in *E. coli* could be stimulated in certain *orc1* mutants.

To reduce the total leaky expression level, the plasmid replication origin along with the selectable marker was replaced so that the plasmid copy number in *E. coli* could be reduced. The resultant plasmid pHK122 bears a p15A *ori* with the *cat* gene and the mutant plasmids pHK123 and pHK124 were successfully constructed, supporting the aforementioned hypothesis regarding the risk of toxicity using pHK118.

### Overexpression of ORC subunits by convenient co-transfection

**Figure 3:**
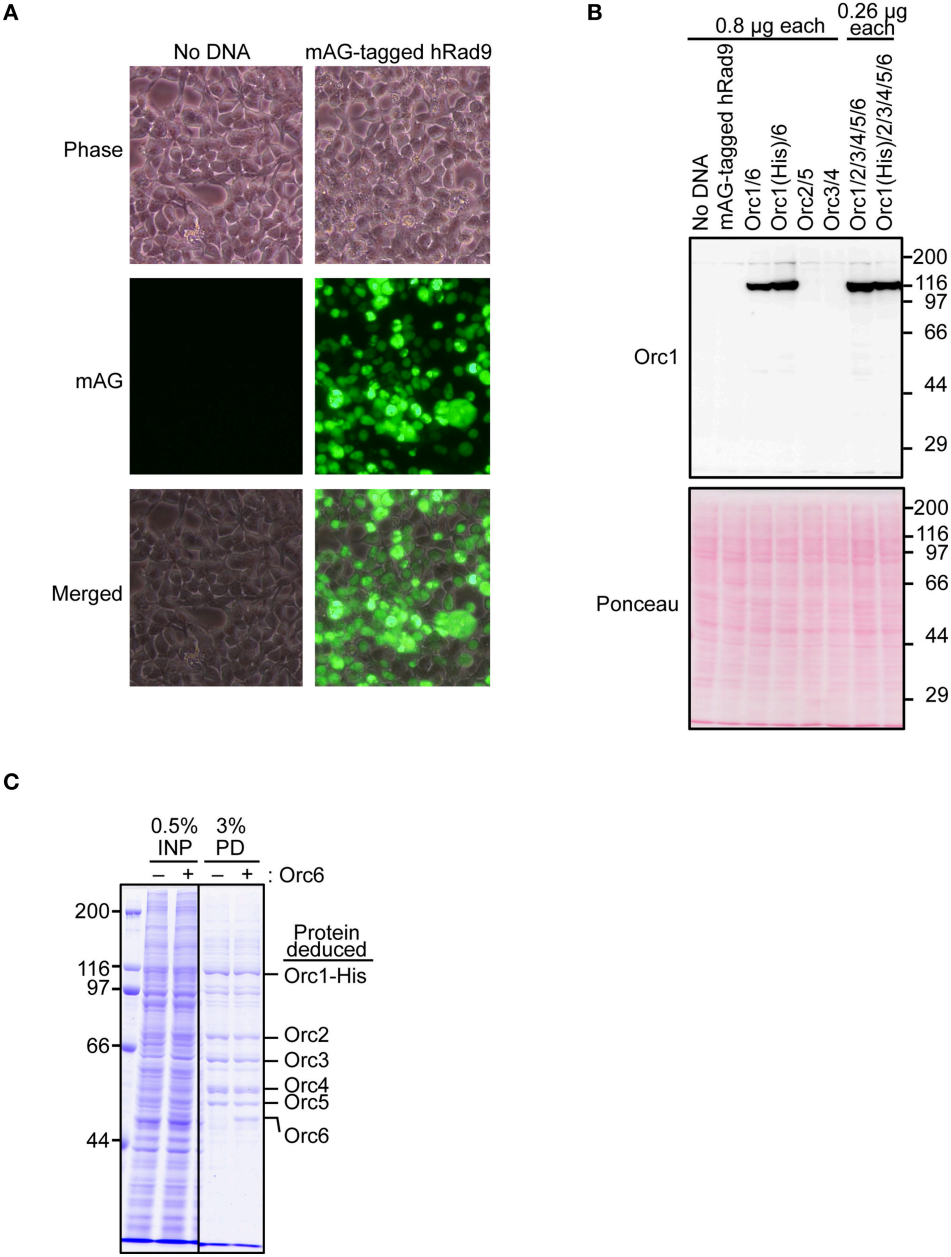
**Co-overexpression of Orc1 and other ORC subunit(s). (A)** Estimation of the transfection efficiency. 293T cells were transfected with a plasmid bearing mAG-tagged hRad9 and incubated for 48 h. Microscopic observation was performed, and phase-contrast and fluorescence images are shown. **(B)** 293T cells were co-transfected with the indicated *ORC* plasmids and incubated for 48 h. Whole cells were lyzed and analyzed by 9% SDS-PAGE, followed by Western blotting using an anti-Orc1 antibody. Ponceau staining was also performed as a loading control. **(C)** Isolation of ORC and Orc1–5 by pulldown. Orc1-His, Orc2, Orc3, Orc4, and Orc5 with (+) or without (–) Orc6 were co-overexpressed. Cleared lysates (input; INP) were subjected to a pulldown assay (PD) and analyzed by 9% SDS-PAGE, followed by Coomassie staining. All lanes originate from the same gel.

To test if Orc1 is overexpressed in 293T cells using the constructed plasmid, a series of transient co-expression experiments were performed. A control experiment using a plasmid expressing mAG-tagged hRad9 indicated that most cells were successfully transfected (Figure 3A). Under this condition, expression of His-tagged Orc1 co-transfected with Orc6 was confirmed by Western blotting (Figure 3B). When intact Orc2/3/4/5/6 plasmids were co-transfected with His-tagged Orc1 plasmid, expression of His-tagged Orc1 was also detected at a level similar to that observed when only His-tagged Orc1 plasmid was transfected. Degradation of Orc1 was not observed. Orc1-His and five major proteins corresponding to Orc2/3/4/5/6 were co-pulled down (Figure 3C), suggesting that all of the ORC subunits were co-overexpressed and formed a complex. Although Uno et al. successfully co-transfected up to three plasmids simultaneously (Uno and Masai, 2011; Uno et al., 2012), our data demonstrated that co-transfection of six plasmids was tolerable for co-overexpression using this system. Hereafter, 0.26 μg of each plasmid per 10-cm plate was used during the course of this study.

### Purification of ORC lacking the Orc6 subunit (Orc1–5)

**Figure 4:**
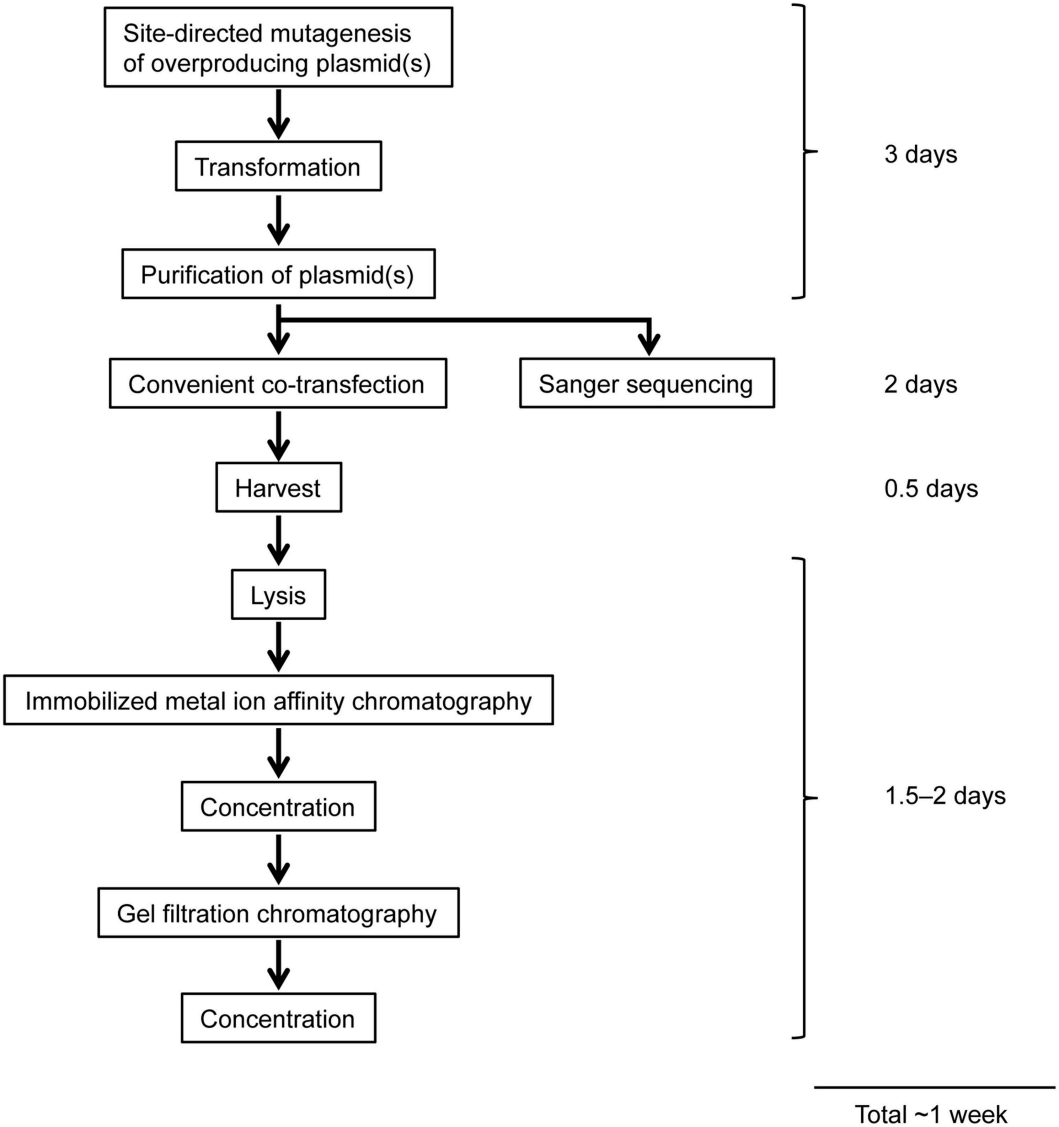
**Workflow of overexpression and purification of mutant ORCs using a mammalian expression system**. See text for details.

To minimize and simplify the column chromatography steps during purification, we first established a purification method of Orc1–5, which takes only 2 days (Figure 4). Orc1/2/3/4/5 form a stable heteropentamer (Lee and Bell, 1997; Chen et al., 2008). The method to purify Orc1–5 from cells using 40 15-cm plates is detailed below.

1. Resuspend the cells in 50 ml of lysis buffer.
2. Add 4500 units of TurboNuclease (Accelagen) and incubate for 30 min.
3. Centrifuge at 15,000 × *g* for 20 min.
4. Transfer the supernatant to 2.5 ml bed volume of MagneHis beads (Promega), prewashed with 50 ml of water, and mix gently for 1 h on a rotation wheel.
5. Wash the magnetic beads five times with 50 ml of wash buffer.
6. Elute the beads four times with 1.7–2.5 ml of elution buffer.
7. Concentrate the peak fractions on a mini SP Sepharose column, followed by a step elution with buffer H/0.4.
8. Load the peak fractions onto a Superdex 200 column equilibrated with buffer H/0.2.
9. Concentrate the peak fractions using another mini SP Sepharose column, divide into aliquots, and snap-freeze in liquid nitrogen.

**Figure 5:**
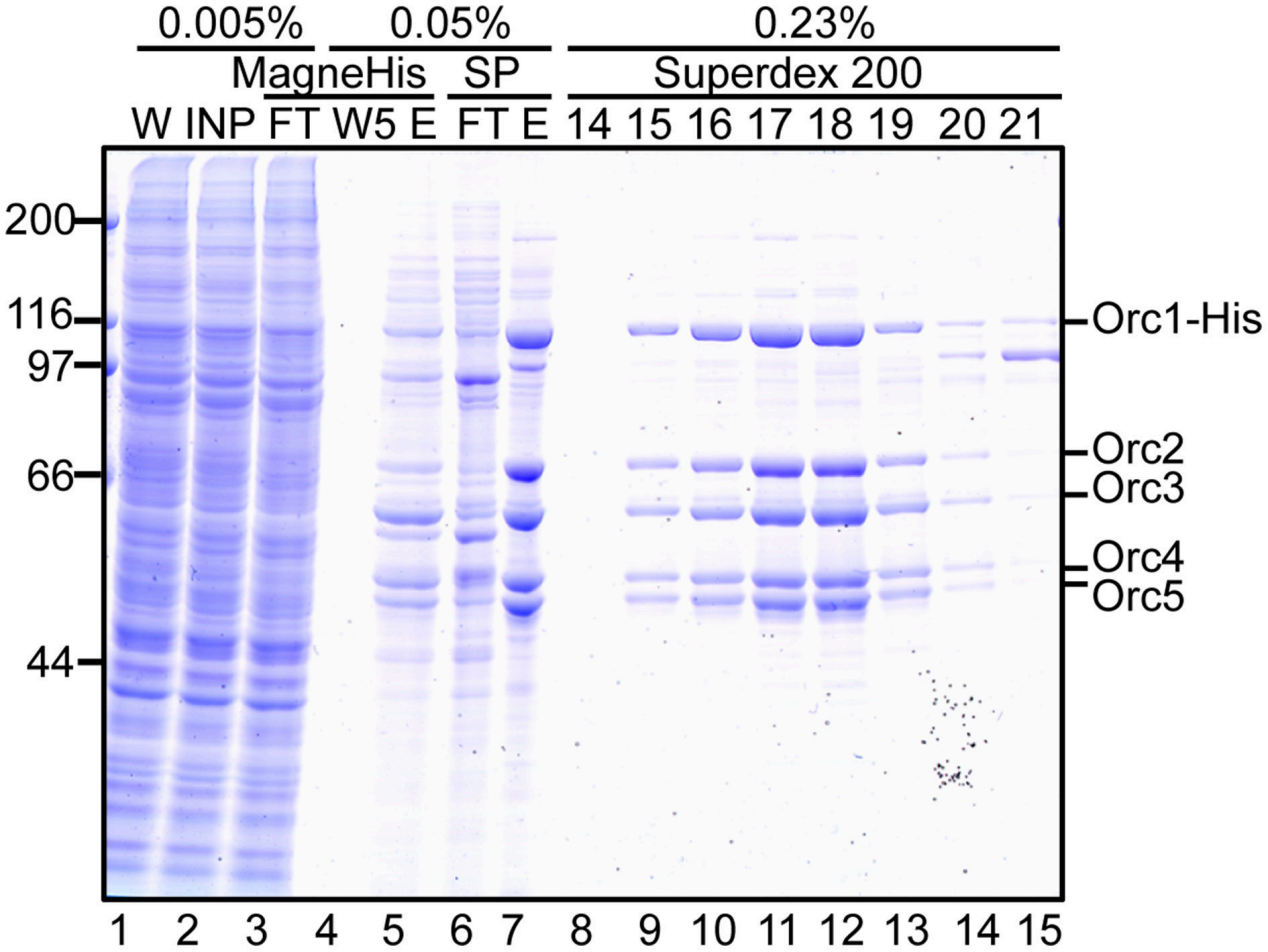
**Purification of Orc1–5**. 293T cells co-overexpressing Orc1-His, Orc2, Orc3, Orc4, and Orc5 were lyzed and fractionated. The indicated volume was taken and analyzed using 9% SDS-PAGE, followed by Coomassie staining. The migration of each ORC subunit is indicated. W, whole cells; INP, input; FT, flow-through; W5, the fifth wash fraction; E, eluate; and 14–21, fraction numbers.

Proteins at each step were monitored by SDS-PAGE (Figure 5). Most cellular proteins were soluble (lanes 1 and 2). Enrichment of a band at ~110 kDa was observed after MagneHis pulldown, corresponding to Orc1-His (106 kDa; lane 5). Some proteins including Orc2/3/4/5 were also enriched (lane 5). Orc1-His and Orc2/3/4/5 were concentrated by SP Sepharose (lane 7 and Table 1A). These proteins co-migrated during gel filtration (lanes 9–14), suggesting that they form a complex. The resultant ORC complex was purified to almost homogeneity. Concentration of the peak fractions yielded ~0.7 mg of protein, which is sufficient for most biochemical applications (Table 1).

**Table 1:** **Purification tables of mutant ORCs**.

**Fraction**	**Step**	**Volume (ml)**	**Concentration (mg/ml)**	**Protein (mg)**	**Yield (%)**
**(A) ORC**					
I.	Lysate	12	28	340^a^	[100]
II.	HisTrap	10	0.84	8.4	2.5
III.	SP Sepharose (first)	1.0	2.6	2.6	0.76
IV.	Superdex 200	6.0	N.D.	N/A	N/A
V.	SP Sepharose (second)	0.65	1.1	0.72	0.21
**(B) Orc1–5**					
I.	Lysate	50	N.D.	N/A^b^	
II.	MagneHis	9.0	1.8	16	[100]
III.	SP Sepharose (first)	1.0	6.5	6.5	41
IV.	Superdex 200	6.0	0.73	4.4	28
V.	SP Sepharose (second)	0.50	4.6	2.3	14
**(C) ORC containing Orc1 K362A**					
I.	Lysate	9.5	10	97^c^	[100]
II.	HisTrap	9.0	0.39	3.5	3.6
III.	SP Sepharose (first)	1.0	1.2	1.2	1.2
IV.	Superdex 200	6.0	0.12	0.73	0.75
V.	SP Sepharose (second)	0.38	1.1	0.40	0.41
**(D) ORC containing Orc1 R367A**					
I.	Lysate	10	16	156^d^	[100]
II.	HisTrap	9.0	0.61	5.5	3.5
III.	SP Sepharose (first)	1.0	2.1	2.1	1.3
IV.	Superdex 200	6.0	0.19	1.2	0.77
V.	SP Sepharose (second)	0.75	1.0	0.74	0.47

a From ~2.4 ml of wet cells (20 15-cm plates).

b From 4 ml of wet cells (40 15-cm plates).

c From ~1 ml of wet cells (20 15-cm plates).

d From ~1.7 ml of wet cells (20 15-cm plates).

### Purification of the ORC hexamer containing Orc1 K362A

**Figure 6:**
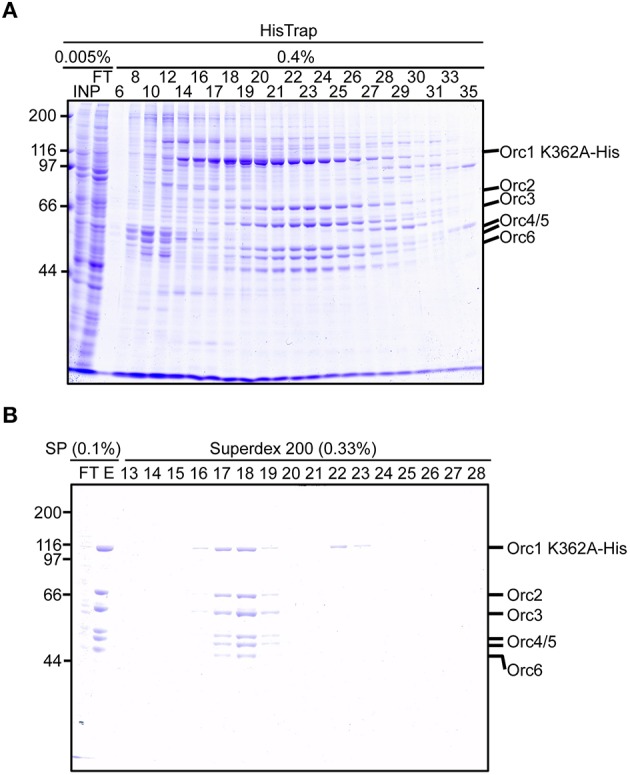
**Purification of ORC containing Orc1 K362A**. 293T cells co-overexpressing Orc1 K362A-His, Orc2, Orc3, Orc4, Orc5, and Orc6 were lyzed and fractionated using HisTrap **(A)** and SP Sepharose and Superdex 200 columns **(B)**. The indicated volume was taken and analyzed using 9% SDS-PAGE, followed by Coomassie staining. The migration of each ORC subunit is indicated.

We next attempted to purify mutant ORC hexamers. This time, we purified ORC containing His-tagged Orc1 K362A from cells using 20 15-cm plates, half the number that was used for Orc1–5. This time we substituted HisTrap column chromatography for MagneHis (Figure 4) to perform a linear gradient elution. The revised method takes only 1 week, including the DNA work such as site-directed mutagenesis. Orc1 K362A-His and Orc1 R367A-His were overexpressed in 293T cells as soluble proteins, similar to wild-type Orc1 (Supplementary Figure 1). When HisTrap column chromatography was performed, Orc1 K362A-His was eluted relatively broadly, peaking at fraction numbers 19–21 (Figure 6A). Some major proteins corresponding to Orc2/3/4/5/6 co-migrated slightly slower, peaking at fraction numbers 23 and 24 (Figure 6A), suggesting that His-tagged Orc1 K362A and His-tagged Orc1 K362A containing Orc2/3/4/5/6 eluted at slightly different imidazole concentrations. Fractions containing all of the ORC subunits were pooled, concentrated, and further fractionated by gel filtration. As expected, His-tagged Orc1-K362A was separated into two fractions, the faster co-migrated with Orc2/3/4/5/6 and the slower eluted alone (Figure 6B). Each band was nearly stoichiometric, suggesting that His-tagged Orc1 K362A as well as Orc2/3/4/5/6 formed a stoichiometric hexamer. Similar results were obtained during preparation of wild-type ORC and ORC containing His-tagged Orc1 R367A; ~0.4–0.7 mg of purified ORC was yielded under these conditions, which is sufficient for typical biochemical assays (Table 1).

### Evaluation of biochemical activities

**Figure 7:**
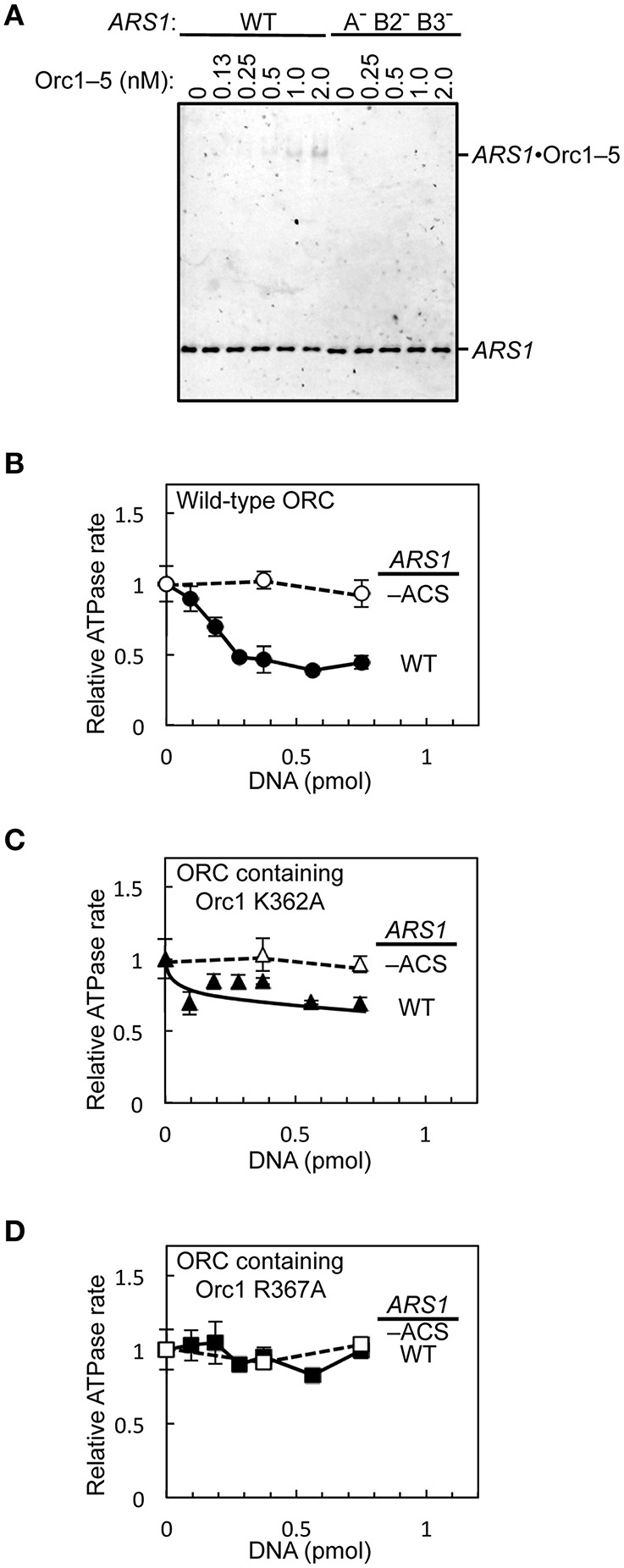
***In vitro* activities of mutant ORC proteins. (A)** The ARS-binding activity of Orc1–5 was examined by an electrophoretic mobility shift assay using Cy5-labeled wild-type (WT) or mutant (A^−^ B2^−^ B3^−^) *ARS1* DNA. **(B–D)** Repression of ORC ATPase activity by *ARS1* DNA in an EOS-dependent manner. WT or mutant (–ACS) *ARS1* DNA was incubated with WT ORC **(B)**, ORC containing Orc1 K362A **(C)**, or ORC containing Orc1 R367A **(D)**, yielding ATPase rates in the absence of DNA of 0.33 **(B)**, 0.28 **(C)**, and 0.25 **(D)** pmol/min/pmol ORC, respectively.

To assess if the purified wild-type and mutant ORCs can be used for downstream applications such as biochemical analyzes, we first performed an electrophoretic mobility shift assay using Orc1–5 and wild-type and mutant *ARS1* DNA. ORC and Orc1–5 bind to ARS at the nanomolar level in *S. cerevisiae* (Speck et al., 2005; Chen et al., 2008). Indeed, an Orc1–5-dependent band shift was seen at concentrations ≤2 nM with wild-type *ARS1,* whereas such shifts were not observed with mutant *ARS1* (Figure 7A).

Next, the effects of *ARS1* DNA on ORC ATPase activity were assessed using ORC containing Orc1 K362A or Orc1 R367A. ORC ATPase activity was repressed by double-stranded DNA in a wild-type ARS sequence-dependent manner (Figure 7B), consistent with a previous finding (Klemm et al., 1997). By contrast, only partial or no significant repression was observed using ORC containing Orc1 K362A and Orc1 R367A, respectively (Figures 7C,D), consistent with the *in vivo* residual ARS-binding activity of Orc1 K362A and Orc1 R367A (Kawakami et al., 2015). These results suggest that mutant ORCs purified using the method reported herein can be used for biochemical applications.

### Versatility of the devised system

In this study, we established a rapid method for the overexpression and purification of mutant ORC hexamers. Compared with previously published methods, the method described in this paper is especially more suitable to purify ORC mutants with multiple mutation sites or lethal effect. Many of the commonly used eukaryotic shuttle vectors bear a ColE1 origin that confers replication at high copy numbers on the shuttle vectors in *E. coli*. Although this property is technically beneficial to yield a large amount of DNA for downstream applications such as cloning and transformation/transfection, it may also cause toxicity in *E. coli* cells when a certain gene is cloned. Replacement of the origin with a lower copy number replicon may overcome such cloning problems in such circumstances. Indeed, Wang and Mullins reported that certain lentivirus-derived sequences can be cloned into a vector bearing a p15A *ori*, but not into vectors bearing a ColE1 *ori* such as pBluescript and pUC (Wang and Mullins, 1995). The combination of 293T cells with the modified expression vector reported in this study will be a powerful tool for future protein overexpression to perform further mutational analyzes of Orc1 and the other ORC subunits, and even purification of proteins other than ORC.

## Author contributions

All the authors conceived and designed the research. HK and TK designed the experiments. HK and EO performed the experiments. HK, EO, and TK analyzed the data. HK and TK wrote the manuscript with critical input from EO and TT.

## Funding

This work was supported by MEXT/JSPS KAKENHI (24870021, 15K18504, 25131714, 25440011, 26114714, and 26291004), the Takeda Science Foundation, the Naito Foundation, the NIG Collaborative Research Program (2011-B1, 2012-B12, and 2013-B1), and the Kyushu University Interdisciplinary Programs in Education and Projects in Research Development.

### Conflict of interest statement

The authors declare that the research was conducted in the absence of any commercial or financial relationships that could be construed as a potential conflict of interest.

## References

[B1] Bell S. P. Kaguni J. M. (2013). Helicase loading at chromosomal origins of replication. Cold Spring Harb. Perspect. Biol. 5:a010124. 10.1101/cshperspect.a010124 23613349 PMC3660832

[B2] Bell S. P. Mitchell J. Leber J. Kobayashi R. Stillman B. (1995). The multidomain structure of Orc1p reveals similarity to regulators of DNA replication and transcriptional silencing. Cell 83, 563–568. 10.1016/0092-8674(95)90096-9 7585959

[B3] Bell S. P. Stillman B. (1992). ATP-dependent recognition of eukaryotic origins of DNA replication by a multiprotein complex. Nature 357, 128–134. 10.1038/357128a0 1579162

[B4] Boos D. Frigola J. Diffley J. F. X. (2012). Activation of the replicative DNA helicase: breaking up is hard to do. Curr. Opin. Cell Biol. 24, 423–430. 10.1016/j.ceb.2012.01.011 22424671

[B5] Bowers J. L. Randell J. C. W. Chen S. Bell S. P. (2004). ATP hydrolysis by ORC catalyzes reiterative Mcm2-7 assembly at a defined origin of replication. Mol. Cell 16, 967–978. 10.1016/j.molcel.2004.11.038 15610739

[B6] Chen Z. Speck C. Wendel P. Tang C. Stillman B. Li H. (2008). The architecture of the DNA replication origin recognition complex in Saccharomyces cerevisiae. Proc. Natl. Acad. Sci. U.S.A. 105, 10326–10331. 10.1073/pnas.0803829105 18647841 PMC2480615

[B7] Coster G. Frigola J. Beuron F. Morris E. P. Diffley J. F. X. (2014). Origin licensing requires ATP binding and hydrolysis by the MCM replicative helicase. Mol. Cell 55, 666–677. 10.1016/j.molcel.2014.06.034 25087873 PMC4157578

[B8] Duncker B. P. Chesnokov I. N. McConkey B. J. (2009). The origin recognition complex protein family. Genome Biol. 10:214. 10.1186/gb-2009-10-3-214 19344485 PMC2690993

[B9] Frigola J. Remus D. Mehanna A. Diffley J. F. X. (2013). ATPase-dependent quality control of DNA replication origin licensing. Nature 495, 339–343. 10.1038/nature11920 23474987 PMC4825857

[B10] Fujita M. Hori Y. Shirahige K. Tsurimoto T. Yoshikawa H. Obuse C. (1998). Cell cycle dependent topological changes of chromosomal replication origins in Saccharomyces cerevisiae. Genes Cells 3, 737–749. 10.1046/j.1365-2443.1998.00226.x 9990508

[B11] Gibson D. G. Young L. Chuang R.-Y. Venter J. C. Hutchison C. A. Smith H. O. (2009). Enzymatic assembly of DNA molecules up to several hundred kilobases. Nat. Methods 6, 343–345. 10.1038/nmeth.1318 19363495

[B12] Hizume K. Yagura M. Araki H. (2013). Concerted interaction between origin recognition complex (ORC), nucleosomes and replication origin DNA ensures stable ORC-origin binding. Genes Cells 18, 764–779. 10.1111/gtc.12073 23795651

[B13] Kawakami H. Katayama T. (2010). DnaA, ORC, and Cdc6: similarity beyond the domains of life and diversity. Biochem. Cell Biol. 88, 49–62. 10.1139/O09-154 20130679

[B14] Kawakami H. Keyamura K. Katayama T. (2005). Formation of an ATP-DnaA-specific initiation complex requires DnaA Arginine 285, a conserved motif in the AAA+ protein family. J. Biol. Chem. 280, 27420–27430. 10.1074/jbc.M502764200 15901724

[B15] Kawakami H. Ohashi E. Kanamoto S. Tsurimoto T. Katayama T. (2015). Specific binding of eukaryotic ORC to DNA replication origins depends on highly conserved basic residues. Sci. Rep. 5, 14929. 10.1038/srep14929 26456755 PMC4601075

[B16] Klemm R. D. Austin R. J. Bell S. P. (1997). Coordinate binding of ATP and origin DNA regulates the ATPase activity of the origin recognition complex. Cell 88, 493–502. 10.1016/S0092-8674(00)81889-9 9038340

[B17] Klemm R. D. Bell S. P. (2001). ATP bound to the origin recognition complex is important for preRC formation. Proc. Natl. Acad. Sci. U.S.A. 98, 8361–8367. 10.1073/pnas.131006898 11459976 PMC37444

[B18] Lee D. G. Bell S. P. (1997). Architecture of the yeast origin recognition complex bound to origins of DNA replication. Mol. Cell. Biol. 17, 7159–7168. 10.1128/MCB.17.12.7159 9372948 PMC232573

[B19] Li H. Stillman B. (2012). The origin recognition complex: a biochemical and structural view. Subcell. Biochem. 62, 37–58. 10.1007/978-94-007-4572-8_3 22918579 PMC3779782

[B20] Li M. Z. Elledge S. J. (2007). Harnessing homologous recombination in vitro to generate recombinant DNA via SLIC. Nat. Methods 4, 251–256. 10.1038/nmeth1010 17293868

[B21] Remus D. Beuron F. Tolun G. Griffith J. D. Morris E. P. Diffley J. F. X. (2009). Concerted loading of Mcm2-7 double hexamers around DNA during DNA replication origin licensing. Cell 139, 719–730. 10.1016/j.cell.2009.10.015 19896182 PMC2804858

[B22] Samel S. A. Fernández-Cid A. Sun J. Riera A. Tognetti S. Herrera M. C.. (2014). A unique DNA entry gate serves for regulated loading of the eukaryotic replicative helicase MCM2-7 onto DNA. Genes Dev. 28, 1653–1666. 10.1101/gad.242404.114 25085418 PMC4117941

[B23] Speck C. Chen Z. Li H. Stillman B. (2005). ATPase-dependent cooperative binding of ORC and Cdc6 to origin DNA. Nat. Struct. Mol. Biol. 12, 965–971. 10.1038/nsmb1002 16228006 PMC2952294

[B24] Speck C. Stillman B. (2007). Cdc6 ATPase activity regulates ORC · Cdc6 stability and the selection of specific DNA sequences as origins of DNA replication. J. Biol. Chem. 282, 11705–11714. 10.1074/jbc.M700399200 17314092 PMC3033201

[B25] Sun J. Evrin C. Samel S. A. Fernández-Cid A. Riera A. Kawakami H.. (2013). Cryo-EM structure of a helicase loading intermediate containing ORC-Cdc6-Cdt1-MCM2-7 bound to DNA. Nat. Struct. Mol. Biol. 20, 944–951. 10.1038/nsmb.2629 23851460 PMC3735830

[B26] Sun J. Kawakami H. Zech J. Speck C. Stillman B. Li H. (2012). Cdc6-induced conformational changes in ORC bound to origin DNA revealed by cryo-electron microscopy. Structure 20, 534–544. 10.1016/j.str.2012.01.011 22405012 PMC3299985

[B27] Tognetti S. Riera A. Speck C. (2015). Switch on the engine: how the eukaryotic replicative helicase MCM2-7 becomes activated. Chromosoma 124, 13–26. 10.1007/s00412-014-0489-2 25308420

[B28] Uno S. Masai H. (2011). Efficient expression and purification of human replication fork-stabilizing factor, Claspin, from mammalian cells: DNA-binding activity and novel protein interactions. Genes Cells 16, 842–856. 10.1111/j.1365-2443.2011.01535.x 21790909

[B29] Uno S. You Z. Masai H. (2012). Purification of replication factors using insect and mammalian cell expression systems. Methods 57, 214–221. 10.1016/j.ymeth.2012.06.016 22800621

[B30] Wang R. F. Mullins J. I. (1995). Mammalian cell/vaccinia virus expression vectors with increased stability of retroviral sequences in Escherichia coli: production of feline immunodeficiency virus envelope protein. Gene 153, 197–202. 10.1016/0378-1119(94)00743-C 7875588

[B31] Yardimci H. Walter J. C. (2014). Prereplication-complex formation: a molecular double take? Nat. Struct. Mol. Biol. 21, 20–25. 10.1038/nsmb.2738 24389553

