# Supplementary material for: Rapid Purification and Characterization of Mutant Origin Recognition Complexes in Saccharomyces cerevisiae
Source: Front Microbiol. 2016 Apr 18;7:521. doi: 10.3389/fmicb.2016.00521 (PMC4834435; doi:10.3389/fmicb.2016.00521)
Supplement: Supplementary file 1 [file Image1.PDF]

Supplementary Material

Rapid Purification and Characterization of Mutant Origin Recognition Complexes in *Saccharomyces cerevisiae*

Hironori Kawakami*, Eiji Ohashi, Toshiki Tsurimoto, and Tsutomu Katayama*

*** Correspondence:**

Hironori Kawakami: kawakami@phar.kyushu-u.ac.jp

Tsutomu Katayama: katayama@phar.kyushu-u.ac.jp

1. Supplementary Figure

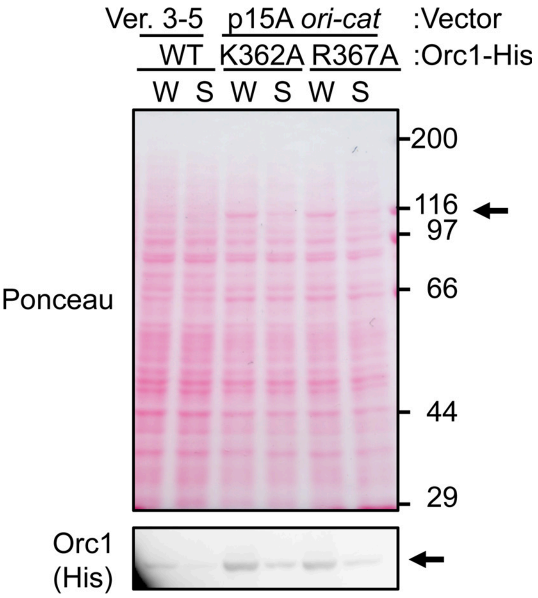

Supplementary Figure 1. Overexpression of Orc1 K362A-His and Orc1 R367A-His. Ponceau staining (upper panel) and Western blotting using an anti-His tag antibody (lower panel) were performed. The migration of Orc1-His is indicated. W, whole cells; and S, cleared lysate.
